# 5-HTT independent effects of fluoxetine on neuroplasticity

**DOI:** 10.1038/s41598-019-42775-w

**Published:** 2019-04-19

**Authors:** Marion J. F. Levy, Fabien Boulle, Michel Boris Emerit, Corinne Poilbout, Harry W. M. Steinbusch, Daniel L. A. Van den Hove, Gunter Kenis, Laurence Lanfumey

**Affiliations:** 1 Institut de Psychiatrie et Neurosciences de Paris, Inserm, U 1266 Paris, France; 2 Université Paris Descartes, UMRS1266 Paris, France; 3 Maastricht University - European Graduate School for Neuroscience EURON, Department of Psychiatry and Neuropsychology, Maastricht, The Netherlands; 4 Laboratory of Translational Neuroscience, Department of Psychiatry, Psychosomatics and Psychotherapy, University of Wuerzburg, Fuechsleinstrasse 15, 97080 Wuerzburg, Germany

**Keywords:** Depression, Neurotrophic factors

## Abstract

Selective serotonin reuptake inhibitors are among the most prescribed antidepressants. Fluoxetine is the lead molecule which exerts its therapeutic effects, at least in part, by promoting neuroplasticity through increased brain-derived neurotrophic factor (BDNF)/tropomyosin-related receptor kinase B (TrkB) signalling. It is unclear however, to which extent the neuroplastic effects of fluoxetine are solely mediated by the inhibition of the serotonin transporter (5-HTT). To answer this question, the effects of fluoxetine on neuroplasticity were analysed in both wild type (WT) and 5-Htt knock-out (KO) mice. Using Western blotting and RT-qPCR approaches, we showed that fluoxetine 10 µM activated BDNF/TrkB signalling pathways in both CD1 and C57BL/6J mouse primary cortical neurons. Interestingly, effects on BDNF signalling were observed in primary cortical neurons from both 5-Htt WT and KO mice. In addition, a 3-week *in vivo* fluoxetine treatment (15 mg/kg/d; i.p.) increased the expression of plasticity genes in brains of both 5-Htt WT and KO mice, and tended to equally enhance hippocampal cell proliferation in both genotypes, without reaching significance. Our results further suggest that fluoxetine-induced neuroplasticity does not solely depend on 5-HTT blockade, but might rely, at least in part, on 5-HTT-independent direct activation of TrkB.

## Introduction

Depression is a complex and heterogeneous neuropsychiatric disorder with a high prevalence and incidence^1^. Despite decades of research, the neurobiological mechanisms underlying its pathogenesis are still poorly understood. Several hypotheses have been put forward, implicating dysregulation of monoamines, stress hormones, neuroplasticity and neuroinflammation^2,3^. However, none of them has emerged as a lead mechanism and probably all are involved, most likely in an intricate fashion, in triggering or maintaining this disease. Imaging studies have nevertheless revealed that structural modifications, such as hippocampal atrophy^4,5^, could occur in recurrent major depression, demonstrating the role of neuroplasticity in this pathology. This was further confirmed in rodent models, reporting impaired neurogenesis (see^6^ for a review), neuron dendrite shrinkage and glial cell loss^7,8^, as well as in postmortem brains, with a decrease in brain-derived neurotrophic factor (BDNF) expression in depressed subjects^9–11^.

Several types of therapies, including psychotherapy, electroconvulsive therapy, deep brain stimulation and pharmacotherapy have shown some efficacy for treating depression. Treatment with available antidepressant molecules still has serious limitations because of their delay of action and lack of efficacy in about one third of patients^12^. One of the most prescribed antidepressants, the selective serotonin reuptake inhibitor (SSRI) fluoxetine, targets the presynaptic serotonin (5-HT) transporter (5-HTT) to increase synaptic 5-HT levels. The beneficial effects of fluoxetine have been shown to be dependent, at least in part, on 5-HT availability since a tryptophan depletion regimen, which decreases brain 5-HT content, leads to a transient depressive relapse in depressed patients during fluoxetine-induced stable remission^13^.

In rodents, chronic fluoxetine treatment is known to promote neuroplasticity, more specifically hippocampal neurogenesis, by inducing cell proliferation, enhancing newborn cell survival, and accelerating neuronal maturation. An increase in both dendritic arborization and length has also been observed in the dentate hippocampus after fluoxetine^14–16^. Fluoxetine is also known to activate several signaling pathways such as MAPK (Mitogen-activated protein kinase)^17^ and CREB (cAMP response element binding protein)^18^ and to modulate BDNF levels^18–21^. BNDF is a neurotrophin that plays a key role in the regulation and maintenance of the central nervous system. It activates three main signaling pathways, i.e. MAPK, phospholipase C-γ (PLCγ) and phosphoinositide 3-kinase (PI3K) pathways through binding with its high affinity receptor: tropomyosin receptor kinase B (TrkB)^22^. TrkB activation promotes cellular survival, proliferation and differentiation as well as synaptic plasticity^23^. Several antidepressants, including fluoxetine, have been shown to phosphorylate TrkB, either in acute or in chronic conditions of treatments^22,24^. Interestingly, data indicate that fluoxetine can directly induce the phosphorylation of TrkB even in the absence of 5-HTT, suggesting that this effect could be independent of 5-HT reuptake blockade^25^ or even of a 5-HT surge, since in 5-Htt null mice, extracellular 5-HT levels measured by microdialysis were unchanged in the hippocampus after fluoxetine administration^26^. In addition, the neurogenic effects of fluoxetine have also been suggested to be partly independent of 5-HTT blockade and have been proposed to involve other targets such as 5-HT receptors^27^. However, the way antidepressant-induced neuroplasticity is related to the 5-HTT site is still unclear.

In this study, we aimed to further dissect the mechanisms of action of fluoxetine on plasticity to better understand to which extent 5-HTT and TrkB/BDNF signaling interact in mediating the neuroplastic effects of this antidepressant. To this aim, we performed both *in vitro* and *in vivo* experiments using wild-type (WT) and 5-Htt constitutive KO^28^ mice to investigate the effects of fluoxetine on markers of neuroplasticity.

## Results

### Acute effects of fluoxetine in primary cortical neurons

#### *Bdnf* mRNA expression

**Figure 1:**
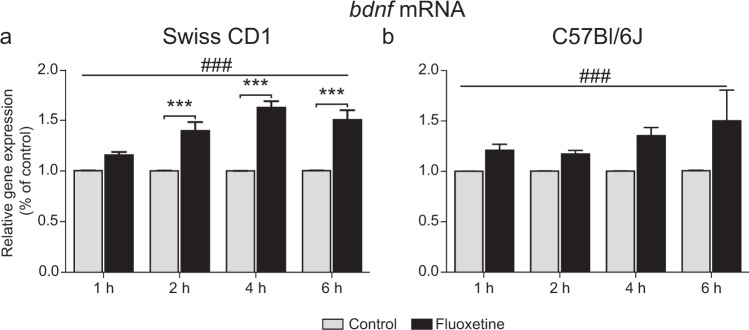
Effects of fluoxetine on *Bdnf* expression in cultured cortical neurons from Swiss CD1 and C57BL/6J mice. Cells were incubated for 1 h, 2 h, 4 h or 6 h with 10 *μ*M of fluoxetine in cultured medium. The expression of *Bdnf* was measured by RT-qPCR. (**a**) In cells issued from Swiss CD1 mouse strain, fluoxetine induced an increase in *Bdnf* mRNA expression 2 h, 4 h and 6 h after incubation. (**b**) In cells issued from C57BL/6J mouse strain, a overall increase of *Bdnf* mRNA expression after fluoxetine incubation was observed. Two-way ANOVA with a Bonferroni post-hoc test. Data are expressed as mean + SEM of n = 3–4. n of 1 is the average of 3 wells. ***p < 0.001 vs. CTL. ^###^p < 0.001, effect of fluoxetine.

In order to validate the acute effect of fluoxetine on *Bdnf* expression, we used primary cortical neurons from Swiss CD1 mouse strain. In these cells, 10 µM fluoxetine significantly increased *Bdnf* expression in a time-dependent manner (two-way ANOVA with Bonferroni post-hoc test: treatment x time interaction: F(3,24) = 7.918, P < 0.001; treatment: F(1,24) = 138.6, P < 0.0001; time: F(3,24) = 7.840 P < 0.001) (Fig. 1a). These data were further confirmed in C57BL/6 J mouse strain. In primary cortical neurons from those mice that expressed 5-HTT as well as TrkB receptors (Supplementary Fig. S1), fluoxetine also increased *Bdnf* expression (two-way ANOVA: treatment effect: F(1,20) = 19.67; P < 0.001), whereas no time effect (F(3,20) = 1.180, P = 0.3424) nor a treatment x time interaction (F(3,20) = 1.133, P = 0.3595) was observed (Fig. 1b).

Of note, *Bdnf* mRNA expression was not modified by 5-HT (1 µM) (2 way ANOVA: treatment effect F(3,16) = 2.928, P = 0.1063), time effect F(3,16) = 2.659, P = 0.0835, treatment × time interaction F(3,16) = 2.720, P = 0.0790) (Supplementary Fig. S2).

#### TrkB, Akt, Erk phosphorylation

**Figure 2:**
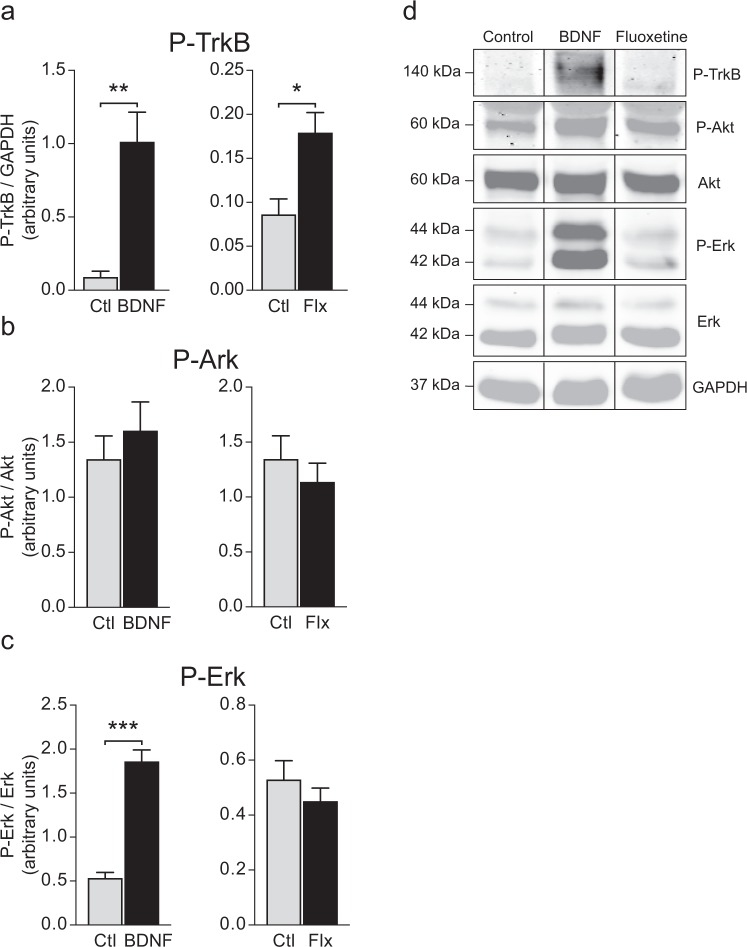
Effects of BDNF and fluoxetine on TrkB activation and downstream signaling. Cells were incubated with 10 nM of BDNF or 10 *μ*M of fluoxetine, 1 h, in cultured medium. Phosphorylation of TrkB (**a**), Akt (**b**) and Erk (**c**) were measured by Western blot. BDNF increased TrkB and Erk phosphorylation and fluoxetine TrkB phosphorylation. (**d**) Representative blots of P-TrkB, P-Akt, Akt, P-Erk, Erk and GAPDH as a reference protein. Full-length blots of these cropped images are presented in Supplementary Fig. S3. The samples derive from the same experiment and blots were processed in parallel. Student t-test. Data are expressed as mean + SEM of n = 6–8. *p < 0.05, **p < 0.01, ***p < 0.001 vs. Ctl. P-TrkB: Phosphorylated TrkB; P-Erk: Phosphorylated Erk; P-Akt: Phosphorylated Akt; Flx: Fluoxetine, Ctl: Control.

In order to determine whether fluoxetine could activate TrkB signaling pathways, primary cortical neurons from C56Bl/6J strain were cultured and treated with fluoxetine (10 µM) or BDNF (1 nM), as a control, for 1 h. As expected, BDNF treatment increased TrkB phosphorylation (t-test: t(11) = 4.078, p = 0.0018), as well as its downstream target, P-Erk (t-test: t(12) = 8.516, p < 0.0001). However, BDNF did not increase P-Akt (t-test t(12) = 0.7816, P = 0.4668). Similarly, treatment with fluoxetine enhanced P-TrkB levels (t-test: t(12) = 2.924, P = 0.0127), but upregulated neither P-Erk (t-test: t(13) = 0.9271, P = 0.3708) nor P-Akt (t-test: t(13) = 0.7440, P = 0.4701) (Fig. 2).

#### Expression of immediate early genes

**Figure 3:**
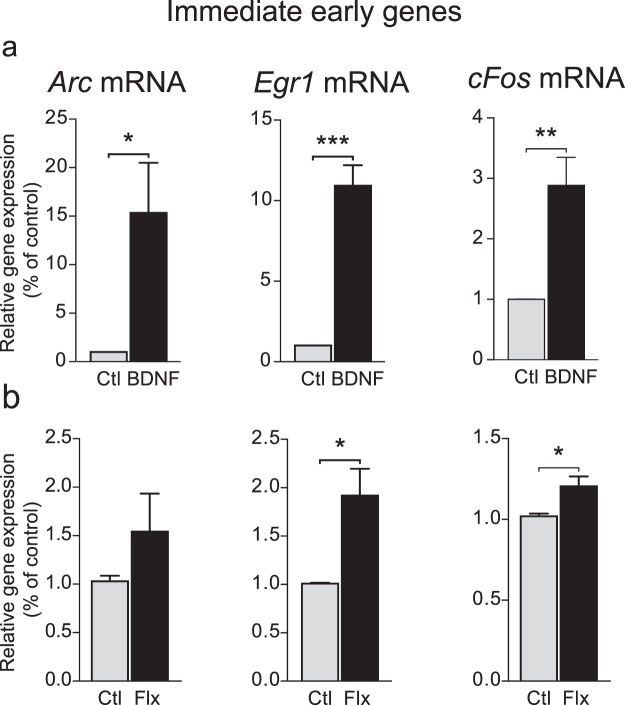
Effects of BDNF and fluoxetine on the expression of immediate early genes in cultured cortical neurons. Cells were incubated with 10 nM of BDNF or 10 *μ*M of fluoxetine, 1 h, in cultured medium. The expression of immediate early genes was measured by RT qPCR. (**a**) BDNF increased *Arc*, *EGR1* and *cFos* mRNA expression (**b**) Fluoxetine also stimulated *EGR1* and *cFos* mRNA expression without modifying that of *Arc* mRNA. Student t test. Data are expressed as mean + SEM of n = 4 (n of 1 is the average of 3 wells). *p < 0.05, **p < 0.01, ***p < 0.001 vs. Ctl. Flx: fluoxetine, Ctl: Control.

To further investigate the role of TrkB in mediating the effects of fluoxetine on plasticity, primary cortical cells were incubated with BDNF or fluoxetine and the expression of several immediate early genes, such as *Arc*, *Egr1* and *cFos*, were measured as markers of plasticity. As presented in Fig. 3a, a Student t-test showed that BDNF treatment (1 nM for 1 hour) induced *Arc*, *Egr1* and *cFos* mRNA expression (*Arc*: t(6) = 2.778, P = 0.0321; *Egr1* t(6) = 7.789, P = 0.0002; *cFos*: t(6) = 4.064, P = 0.0066). When cells were incubated with fluoxetine (10 µM), a Student t-test also revealed an effect of fluoxetine on *Egr1* and *cFos* mRNA expression (*Egr1*: t(6) = 3.275, P = 0.0169; *cFos*: t(6) = 3.026, P < 0.0232), while *Arc* mRNA expression was not increased (t-test: t(6) = 1.299, P = 0.2417) (Fig. 3b).

#### Acute effects of fluoxetine in 5-Htt KO primary cortical neurons

**Figure 4:**
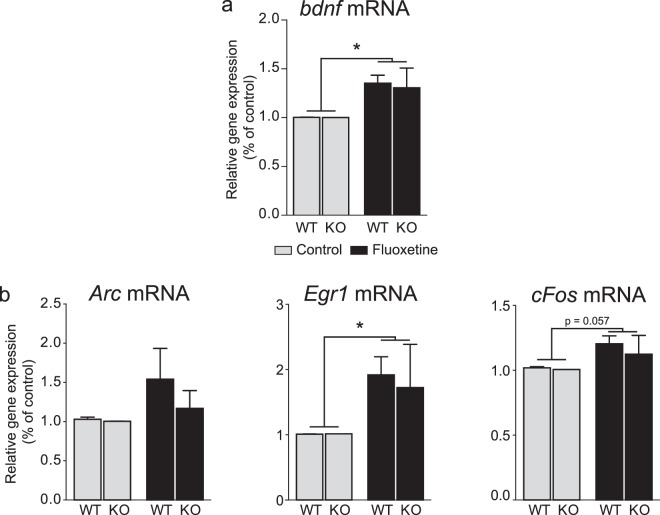
Effects of fluoxetine on *Bdnf*, *Arc*, *Egr1*, *cFos* gene expression in cultured cortical neurons from WT and 5-Htt KO mice. Cells from WT and 5-Htt KO mice were incubated with fluoxetine 10 *μ*M, for 1 h (**a**) or 4 h (**b**), in cultured medium. The expression of *Bdnf*, *Arc*, *Egr1* and *cFos* was measured by RT-qPCR. Data showed an increase in *Bdnf* and *Egr1* expression with fluoxetine in cells from both WT and 5-Htt KO mice. Two-way ANOVA. Data are expressed as mean + SEM of n = 3–4 (n of 1 is the average of 3 wells). *p < 0.05, vs. Control. WT: wild-type mice; KO: 5-Htt knock-out mice. Data for WT are those reported in Figs 1 and 3.

In order to examine the role of 5-HTT in mediating the plasticity-related effects of fluoxetine, we replicated the aforementioned experiments in primary cortical neurons from 5-Htt KO mice. 10 μM fluoxetine induced an increase in *Bdnf* mRNA expression in cells from both WT and 5-Htt KO mice after a 4 h incubation (two-way ANOVA: effect of treatment: F(1,8) = 9.110, P = 0.0166). Neither an effect of genotype, nor an interaction between genotype and treatment was observed (two-way ANOVA, effect of genotype: F(1,8) = 0.05434, P = 0.8215; treatment x genotype interaction: F(1,8) = 0.04610, P = 0.8354), evidencing that fluoxetine could exert its effects on *Bdnf* mRNA expression even in the absence of 5-HTT (Fig. 4a).

Regarding early gene induction, a two way ANOVA showed a fluoxetine (10 µM, 1 h) effect on *Egr1* expression (F(1,10) = 6.317, P = 0.0307), with a tendency of the antidepressant to increase that of *cFos* (F(1,10) = 4.585, P = 0.0579) without affecting that of *Arc* (F(1,10) = 1.803, P = 0.2090). There was no effect of genotype (*Arc*: F(1,10) = 0.6246, P = 0.4477; *Egr1*: F(1,10) = 0.08641, P = 0.7748; *cFos*: F(1,10) = 0.4333, P = 0.5252) and no treatment × genotype interaction (*Arc*: F(1,10) = 0.4748, P = 0.5065; *Egr1*: F(1,10) = 0.09487, P = 0.7644; *cFos*: F(1,10) = 0.2259, P = 0.6448) (Fig. 4b).

### Chronic effects of fluoxetine on plasticity in WT and 5-Htt KO mice

#### *BDNF*/*TrkB* signaling-related gene expression

In order to support the *in vitro* data, fluoxetine effects were also investigated in *in vivo* conditions using WT and 5-Htt KO mice. Mice were injected with saline or fluoxetine (15 mg/kg/d i.p.) for three weeks. RT-qPCR experiments were performed on both the hippocampus and the frontal cortex of the left hemisphere of each mouse.

**Figure 5:**
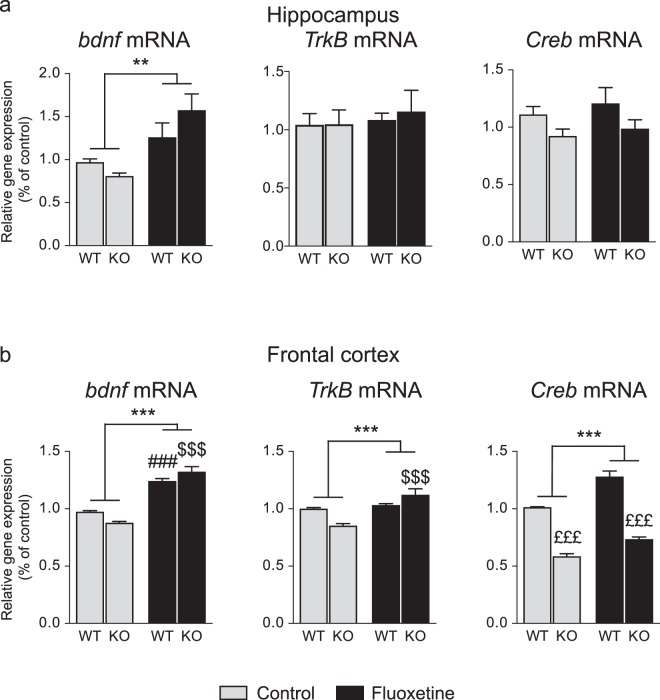
Effects of chronic treatment of fluoxetine on expression of plasticity-related genes in WT and 5-Htt KO mice. WT and 5-Htt KO male mice were injected with fluoxetine (15 mg/kg, daily) for 21 days. Hippocampus and frontal cortex from the right hemisphere were collected and plasticity-related gene expression was measured by RT-qPCR. (**a**) In the hippocampus, data showed an increase of *Bdnf* mRNA expression in fluoxetine-treated KO mice. No significant change was observed in that of *TrkB* and *Creb*. (**b**) In the frontal cortex data showed that *Bdnf*, *Creb* and *TrkB* mRNA expressions were increased in fluoxetine-treated mice for both WT and KO mice. Two-way ANOVA with a Bonferroni post-hoc test. Data are expressed as mean + SEM of n = 6–12. *p < 0.05, **p < 0.01, ***p < 0.001 vs. Control. ^###^p < 0.001 vs WT-Control; ^$$$^p < 0.001 vs KO-Control; ^£££^p < 0.001 vs WT. WT: Wild type mice; KO: 5-Htt knock out mice.

In the hippocampus, a two-way ANOVA revealed a treatment effect with an increase in *Bdnf* mRNA expression (F(1,35) = 11.87, P = 0.0015), but no effect of genotype (F(1,35) = 0.255, P = 0.616) nor a treatment × genotype interaction (F(1,35) = 2.42, P = 0.1288). Chronic fluoxetine did not modify *Trkb* mRNA expression (two-way ANOVA: treatment: F(1,23) = 0.3263, P = 0.5734; genotype: F(1,23) = 0.08824, P = 0.7691; treatment x genotype interaction: F(1,23) = 0.06707, P = 0.7980), nor that of *Creb* (two-way ANOVA: effect of treatment: F(1,34) = 0.5533, P = 0.4621; effect of genotype: F(1,34) = 3.662, P = 0.0641; treatment × genotype interaction: F(1,35) = 0.02366, P = 0.8787) (Fig. 5a).

In the frontal cortex, fluoxetine upregulated *Bdnf*, *TrkB*, and *Creb* mRNA expression in both fluoxetine-treated WT and 5-Htt KO mice (two-way ANOVA *Bdnf*: F(1,35) = 138.0, P < 0.0001; *Trkb*: F(1,24) = 20.38 p < 0.0001; *Creb*: F(1,37) = 32.53, P < 0.0001). *Bdnf* and *TrkB* mRNA expression also showed a treatment × genotype interaction (*Bdnf*: F(1,35) = 8.488, P = 0.0062, *TrkB*: F(1,24) = 12.64 P = 0.0016). A Bonferroni post-hoc test revealed a higher mRNA expression of *Bdnf* and *TrkB* in the fluoxetine-treated 5-Htt KO mice (*Bdnf*: control vs fluoxetine: WT mice: t(20) = 6.709, P < 0.001; 5-Htt KO mice: t(15) = 9.795, P < 0.001; *TrkB*: control vs fluoxetine: WT mice t(13) = 0.7041 P > 0.05; 5-Htt KO mice t(11) = 5.510, P < 0.001). There was no genotype effect (*Bdnf*: F(1,35) = 0.05875, P = 0.8099; *TrkB*: F(1,24) = 0.7872, p < 0.3838). Regarding *Creb* mRNA expression, in addition to a treatment effect, a genotype effect was also observed with a global decreased expression in 5-Htt KO mice (two-Way ANOVA: *Creb*: F(1,37) = 174.3 P < 0.0001), without treatment x genotype interaction (two-way ANOVA: *Creb*: F(1,37) = 2.503 P < 0.1221) (Fig. 5b).

#### *BDNF*/*TrkB* signaling-related protein activation

To further study TrkB signaling at protein level, we performed Western blots to analyze plasticity-related protein activation. Unexpectedly, a 3-week treatment with fluoxetine up-regulated neither TrkB nor CREB phosphorylation in the hippocampus and the frontal cortex, regardless of the genotype (two-way ANOVA hippocampus: P-TrkB F(1,14) = 0.1881, P = 0.671; P-CREB F(1,14) = 2.218 P = 0.1586; frontal cortex: P-TrkB F(1,23) = 0.2616, P = 0.6139; P-CREB F(1,23) = 0.3038, P = 0.5868) (Table 1). Furthermore, two-way ANOVA revealed neither a genotype effect (hippocampus: P-TrkB F(1,14) = 0.3014, P = 0.5917; P-CREB F(1,14) = 0.4119 P = 0.5314; frontal cortex: P-TrkB F(1,23) = 0.1205, P = 0.7316; P-CREB F(1,23) = 1.234 P = 0.2782), nor a treatment × genotype interaction (hippocampus: P-TrkB F(1,14) = 1.019, P = 0.3298; P-CREB F(1,14) = 2.070 P = 0.1722; frontal cortex: P-TrkB F(1,23) = 0.6963, P = 0.4126; P-CREB F(1,23) = 0.02648 P = 0.8722).

**Table 1:** Effects of chronic treatment of fluoxetine on the activation of plasticity-related proteins in WT and 5-Htt KO mice.

	WT		KO	
	Saline	Fluoxetine	Saline	Fluoxetine
	**Hippocampus**			
P-TrkB/TrkB	100.0 +/− 19.95	94.08 +/− 5.343	95.26 +/− 12.77	110.1 +/− 9.333
P-CREB/CREB	100.0 +/− 9.951	99.29 +/− 12.30	129.3 +/− 12.80	88.04 +/−19.60
	**Frontal Cortex**			
P-TrkB/TrkB	100.0 +/− 13.53	71.22 +/−21.56	74.74 +/− 25.69	81.64 +/− 23.73
P-CREB/CREB	100.0 +/− 18.86	105.6 +/− 10.78	81.46 +/− 9.775	91.85 +/− 15.48

Male C57Bl/6J WT and 5-Htt KO mice were injected with fluoxetine (15 mg/kg) daily for 21 days. Hippocampus and frontal cortex from the right hemisphere were collected and plasticity-related protein activation was measured by Western blot. In the hippocampus, results indicated no change in TrkB activation and CREB activation both in the fluoxetine treated WT and KO mice. In the frontal cortex, results indicated no change in TrkB activation and CREB activation both in the fluoxetine treated WT and KO mice. Two-way ANOVA. Data represent ratios of intensity values of the phosphorylated versus total protein with the saline condition as the reference, and are expressed as mean +/− SEM of n = 3–7. WT: Wild type mice; KO: 5-Htt knock out mice.

#### Hippocampal cell proliferation

**Figure 6:**
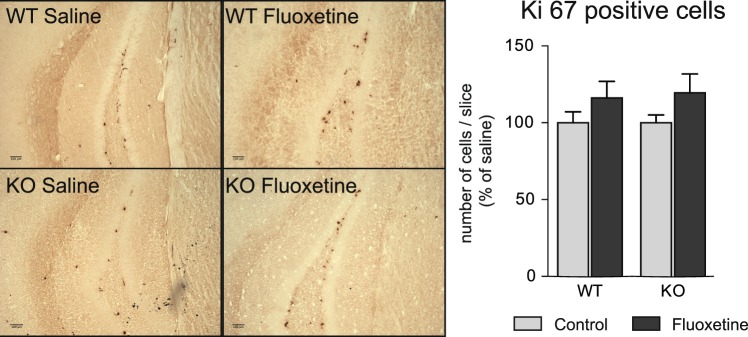
Effects of chronic treatment with fluoxetine on cell proliferation in 5-Htt WT and KO mice. Male 5-Htt WT and KO mice were injected with fluoxetine (15 mg/kg, daily) for 21 days. Ki67-positive cells were stained by immunohistochemistry in the hippocampus. Results indicated no significant effect of the treatment. Multiple t tests. Data are expressed as mean + SEM of n = 4–7. WT: Wild type mice; KO: 5-Htt knock out mice.

We used the right hemisphere of the same mice to study fluoxetine-induced plasticity at a cellular level. To this aim, we investigated the effect of fluoxetine on the number of proliferative cells within the dentate gyrus of WT and 5-Htt KO mice. Although fluoxetine treatment did not have a significant effect, it nevertheless tended to increase Ki67 cell labelling in a similar proportion in KO and in WT mice (+19.4 ± 12.3%, vs +14.0 ± 11.2%, KO and WT respectively, mean ± SEM, n = 4–7, ns, multiple t tests) (Fig. 6).

## Discussion

Although fluoxetine is one of the most prescribed antidepressants, its effects on neuroplasticity, which probably contribute to its therapeutic efficacy^14,22^, are still not fully understood. Together with its ability to increase the extracellular concentration of 5-HT by inhibiting 5-HTT, fluoxetine also exerts anti-inflammatory, anti-apoptotic and antioxidant actions via different mechanisms of action^29^. Regarding neuroplasticity^30^, it was however unclear whether this effect was mediated solely by 5-HTT inhibition, especially since it has been shown that it could directly induce the phosphorylation of TrkB even in the absence of 5-HTT^25^. In the present study, we showed that the effects of fluoxetine on neuroplasticity markers were independent of 5-HTT inhibition. In primary cortical neuron cultures, fluoxetine activated TrkB and induced expression of BDNF-related immediate early genes. In addition, fluoxetine upregulated *Bdnf, Egr1* and *cFos* gene expression even in the absence of 5-HTT. *In vivo*, fluoxetine induced *Bdnf* and other plasticity-related gene expression activation in both WT and 5-Htt-deficient mice, further evidencing a 5-HTT-independent action of this antidepressant on BDNF/TrkB signaling.

The present data further demonstrate that fluoxetine can promote plasticity in both primary cell cultures and after chronic *in vivo* treatment. Although widely investigated in *in vivo* conditions^31–33^, the *in vitro* effects of fluoxetine on *Bdnf* mRNA expression have been reported in only a few studies. All showed that fluoxetine can activate *Bdnf* expression, in control conditions, as well as in stressful environments, such as under toxic conditions induced by B27 deprivation in primary rat hippocampal cultures^34^ or in corticosterone-exposed PC12 cells^35^. We further confirmed and complemented those data showing that fluoxetine induces *Bdnf* mRNA expression and also activates immediate early gene expression, such as *Egr1* and *Cfos*, and stimulates TrkB phosphorylation in C57Bl/6J primary cortical cell cultures. Some studies have reported no effect of tricyclic antidepressants, amitryptiline and imipramine, on TrkB phosphorylation, neither in hippocampal or cortical cell cultures, nor in fibroblasts expressing catalytic TrkB receptors^25^. However, neither the time of application (15 min) nor the antidepressants (amitryptiline and imipramine) were similar to our conditions, hindering any direct comparison between these studies. Besides, in our hands, fluoxetine activated neither MAPK pathway (Erk) nor Akt phosphorylation, in contrast to BDNF. This might be due to the delayed action of fluoxetine on the downstream signaling pathways, only occurring after the initial TrkB phosphorylation. In addition, the high affinity of BDNF for TrkB probably allows the peptide to initiate this pathway with a better efficacy than fluoxetine. Besides, Akt and Erk activation on hippocampal primary cells has been shown to occur only after 4–5 days of fluoxetine incubation, indicating the requirement of a prolonged stimulation with an antidepressant to activate these pathways^36^.

However, *Bdnf* activation took place between 2 and 6 hours after the beginning of fluoxetine exposure, while both early gene expression and TrkB phosphorylation occurred as soon as 1 h after incubation, at a time where BDNF levels were not yet increased. The magnitude of TrkB phosphorylation expression was lower after fluoxetine than after BDNF incubation probably because BDNF directly activated TrkB receptors, while the effect of fluoxetine occurred probably through its transactivation, further suggesting that TrkB can be activated independently from neurotrophins, as proposed by Rantamaki *et al*.^25^. This last study indeed showed a rapid action of imipramine (30 min, 30 mg/kg), on TrkB phosphorylation both in the presence and absence of BDNF, suggesting that antidepressants do not require BDNF to activate TrkB.

Interestingly, fluoxetine effects on *Bdnf* and *Egr1* mRNA expression were still observed in primary cortical cell cultures where the main target of SSRIs, the 5-HTT, was inactivated. This effect strongly suggests that the ability of fluoxetine to upregulate *Bndf* and *Egr1* mRNA expression *in vitro* was, at least in part and in our conditions, 5-HTT-independent. Although not significant, fluoxetine also tended to activate *cFos* mRNA expression. These data were also supported by those obtained in *in vivo* conditions. Using adult WT and 5-Htt KO mice, chronic fluoxetine treatment induced neurotrophic effects that were independent of the genotype. Although in the hippocampus fluoxetine effect was limited to *Bdnf* expression, in the PFC, the 3 week-fluoxetine treatment upregulated *Bdnf, TrkB and Creb* mRNA in both WT and KO mice, and at least for *Bdnf* and *TrkB*, fluoxetine effects appeared to be greater in absence than in presence of 5-HTT. Whether the 5-HTT–independent mechanisms involved in the effects of fluoxetine were up regulated in the absence of the uptake site is a question to be addressed, but however, these data do not endorse those reported in 5-Htt mutated rats, where *Bdnf* expression was much lower than in WT, and where chronic fluoxetine was not able to significantly up-regulated it^37^. Nevertheless, our data indicated a more efficient effect of this antidepressant on BDNF/TrkB signaling in the mouse frontal cortex, stressing again the role of this structure in the pathogenesis of depression, as supported by other studies^38^.

Regarding protein levels, chronic fluoxetine failed to enhance plasticity-related proteins in both the PFC and the hippocampus, whatever the genotype, in contrast to the reported acute effects of this antidepressant on TrkB activation^22^, even in 5-Htt-deficient mice^25^. Whether protein phosphorylation might be regulated through post-translational mechanisms is a question to be further addressed.

Finally, proliferation studies also indicated a similar tendency of fluoxetine to increase the number of proliferative cells in 5-Htt KO and WT mice, further supporting the 5-HTT independency of fluoxetine on plasticity. The rather low efficiency of fluoxetine-induced proliferation we found in the present study was not unexpected, since this has also been reported by other studies, especially those performed in well controlled conditions, i.e., in the absence of any aversive environment such as stress^20,21,39^. In addition, fluoxetine effects on cell proliferation has also been reported to be dependent on the duration of the treatment as well as the dose and the method used to treat the animals^40–42^. Conversely, in 5-Htt mutated mice in which only the SSRI binding site was inactivated, fluoxetine has been shown to lose its proliferative properties, suggesting, that, *in vivo*, potentiated 5-HT signaling was essential to the ability of SSRIs to stimulate subgranular stem cell expansion^43^, in discrepancy with our data.

Yet, in a null 5-Htt mutant model, in both *in vitro* and *in vivo* conditions, whatever the marker to be studied, fluoxetine induced similar effects in 5-Htt KO mice than in WT paired controls, suggesting that in the absence of 5-HTT, fluoxetine was still able to act on neuroplasticity. Whether these effects are linked to 5-HT, or related to a direct activation of BDNF/TkrB complex, or to other pathways is subject of future investigation.

The notion that antidepressants can transactivate brain TrkB receptors independently of monoamine reuptake blockade has been proposed some years ago^22,25^. TrkB receptors have been demonstrated to be transactivated by e.g. glucocorticoids receptors^44^ or adenosine receptors^45^. Our data mostly support and extend this hypothesis by proposing that also in cultured cortical primary cells, fluoxetine enhances *Bdnf* gene expression. These results highlight the potential direct effect of the BDNF/TrkB signaling pathway in the mechanism of action of antidepressants^46^ and might further indicate that TrkB is a valuable target to treat depression^23,47^.

Altogether, these data showed that fluoxetine might induce neuroplastic effects through 5-HTT-independent activation of BDNF/TrkB complex signaling. Whether these effects are mediated through a direct transactivation of the TrkB receptor, and how BDNF/TrkB signaling, 5-HT, and/or other processes and pathways are interconnected in regulating fluoxetine-induced neuroplasticity awaits further research.

## Methods

All procedures concerning animal care and treatments were carried out in accordance with the protocols approved by the ethical committee # C2EA -05 Charles Darwin for the use of experimental animals and were licensed by the Directorate General for Research and Innovation (French Ministère de l’Enseignement Supérieur et de la Recherche), under protocol authorization # 00966.02.

### Animals

CD1 and C57BL/6J mice were purchased from Janvier Laboratories (Saint Berthevin, France). Experiments were performed using homozygous 5-Htt−/− (KO), and 5-Htt+/+ (WT) littermates born from heterozygous (+/−) mutants, bred on a C57Bl/6J background^28^. Genotyping was performed as previously described in detail^48^. Experiments were started at 2–3 months of age when body weight in each genotype equally ranged between 20 and 25 g. After weaning, males were housed in groups of 4–6 animals per cage and maintained under standard laboratory conditions (22 ± 1 °C, 60% relative humidity, 12–12 hour light-dark cycle, food and water *ad libitum*).

### Primary cell cultures

We used cells from two different mouse strains, Swiss CD1 and C57Bl/6J. The first data were obtained in CD1 mice in which we could collect several embryos. When the experimental conditions were set up, we performed the next experiments on the C57BL/6J mouse strain, on which the 5-Htt constitutive knock down was performed^28^.

Embryos from pregnant Swiss CD1 strain, as well as from 5-Htt WT and KO mice on a C57BL/6J strain were isolated at embryonic day 16. The dissection of the cortices was performed in Neurobasal medium (Gibco, Thermo Fisher Scientific, Courtaboeuf, France), and, after collection, the tissues were maintained in Hank’s Balanced Salt Solution (HBSS) (Gibco, Thermo Fisher Scientific) until trypsinization. Dissociation of the tissues was first achieved with trypsin (Sigma Aldrich, Saint Quentin Fallavier, France), and then terminated with a mechanical dissociation with a glass pipette. Cells were counted and plated on 6-well cultures plates coated with poly-D-lysine (Sigma Aldrich) at a density of 1000 cells/mm^2^ for quantitative reverse transcriptase polymerase chain reaction (qRT-PCR) purposes, or on poly-D-lysine-coated 25 cc flasks at a density of 1600 cells/mm^2^ for Western blotting. Cells were cultured in complete Neurobasal medium supplemented with B27 (Gibco, Thermo Fisher Scientific), containing 0.5 mM L-glutamine, 10 U/ml penicillin G, and 10 mg/ml streptomycin (Gibco, Thermo Fisher Scientific), and used for experiments on the fifth day *in vitro* (DIV5).

### Drug treatment and brain collection

Ten-week-old WT and KO male mice were injected (i.p.) daily with fluoxetine (15 mg/kg, Sigma Aldrich) or saline solution (0,9% NaCl) for 3 weeks according to published work^49,50^. Mice were sacrificed by cervical dislocation 24 hours after the last injection. The hippocampus and frontal cortex from the left hemisphere were collected and immediately frozen in liquid nitrogen and stored at −80 °C until use. The right hemisphere was frozen in isopentane at −30 °C, and subsequently stored at −80 °C until further use.

### Quantitative reverse transcriptase PCR (qRT-PCR)

mRNA expression of *Bdnf*, *Arc*, *Egr1* and *cFos* gene was assessed *in vitro* and that of *Bdnf TrkB* and *Creb* gene awas analyzed *in vivo* (see supplementary Table S1 for the sequences). Cells were incubated in the above-mentioned medium supplemented with fluoxetine (10 µM)^34^ BDNF (1 nM; Peprotech, Neuilly-Sur-Seine, France)^51^ or vehicle (milli-Q water) at doses previously published^34,51^. Total mRNA extraction was performed using NucleoSpin RNA II kit (Macherey-Nagel, Hoerdt, France). TRIzol RNA Isolation Reagent (Invitrogen, CA, USA) was used to isolate the total RNA from the hippocampus and the frontal cortex of WT and KO mice. cDNA synthesis was performed using a High Capacity cDNA Reverse Transcription kit (Applied Biosystems, Courtaboeuf, France) according to the manufacturer’s protocol. Amplification was performed with KAPA SYBR FAST qPCR kit (KAPABIOSYSTEMS, Clinisciences, Nanterre, France) using the 7300 Real Time PCR System (Applied Biosystems). The sequences of primers used are indicated in Supplementary Table 1. RT-qPCR conditions involved 40 cycles in a fixed sequence, i.e. 95 °C for 15 s, 62 °C for 30 s, and 72 °C for 30 s. The 2^ΔΔCT^ (Delta-Delta Comparative Threshold) method was used to normalize the fold change in gene expression. Gene expression was normalized using *Actb* as a reference gene that was shown to be stable over the groups.

### Western blots

In *in vitro* study, protein expression levels for TrkB, P-TrkB, Akt, P-Akt, Erk, P-Erk and the reference protein GAPDH were assessed by Western blotting (see Supplementary Table S2 for information on supplier and dilution). Cells from WT mice were incubated for 1 h with BDNF (1 nM), fluoxetine (10 µM) or vehicle. After incubation, cells were washed with ice cold PBS (Gibco, Thermo Fisher Scientific) and proteins were extracted using an ice-cold lysis buffer (50 mM Tris-HCl, pH 7.4, 1% Triton X-100, 150 mM NaCl; phosphatase inhibitors: Na_3_VO_4_ (1 mM) and NaF (10 nM); protease inhibitor cocktail [Roche, Meylan, France]). The lysate was then centrifuged for 15 min at 14,000 g and the supernatant was collected. The protein concentration was determined by the Lowry protein assay (DC protein assay, Bio-Rad Hercules, USA). The extract was mixed with sample buffer (5:1, v/v) containing 188 mM trisHCl, 6% SDS, 30% glycerol, 15% β-mercaptoethanol, and 0.01% bromophenol blue. The mix was heated for 5 minutes at 95 °C. 20 µg of proteins were then separated in a 10% SDS-PAGE gel and transferred on a nitrocellulose membrane for 1 h at 100 mV. The membranes were blocked with 1:1 v/v Odyssey blocking buffer (Li-Cor Biosciences, NE, USA,) in PBS for 1 h at room temperature (RT), incubated with the primary antibody overnight at 4 °C and, subsequently, with secondary antibodies (see Supplementary Table S2 for details) for 1 h at RT. The membranes were scanned using the Odyssey system (Odyssey, Li-cor Biosciences, Leusden NL) and analyzed with ImageJ.

In the *in vivo* study, proteins were extracted from WT and KO mice using TRIzol© RNA isolation reagents (Invitrogen,) according to a previous described protocol^52^. Briefly, the proteins were washed and dissolved in a 1:1 (v/v) mixture of 8 M urea and 1% SDS and sonicated. Protease and phosphatase inhibitor tablets were added to the mixture (Roche, Switzerland). Protein concentration was determined by the Pierce^TM^ BCA Protein Assay Kit (Thermo Fisher Scientific). Extracts were mixed with NuPAGE™ LDS Sample Buffer (Thermo Fisher Scientific). The mix was heated for 10 minutes at 95 °C. 30 μg of protein was then separated using a gradient 4–12% gel (NuPAGE™ 4–12% Bis-Tris Protein Gels, Thermo Fisher Scientific) and transferred on a nitrocellulose membrane with the iBlot® Dry Blotting System (Thermo Fisher Scientific) for 7 min. Membranes were blocked (1:1 v/v Odyssey blocking buffer, Li-Cor Biosciences, NE, USA, in TBS) for 1 h at RT, incubated with the primary antibody overnight at 4 °C and with secondary antibodies for 1 h at RT. The dilution and reference of the antibodies are indicated in Supplementary Table 2.

The membranes were finally scanned using the Odyssey CLx system (Odyssey, Li-Cor Biosciences, NE, USA) and analyzed with ImageJ.

### Immunohistochemistry

Brains were cut into 20-μm-thick coronal sections. Frozen hippocampal sections (from Bregma −1.34 to Bregma −3.20) were fixated with 4% paraformaldehyde (in 0,1 M PBS) for 10 min at 4 °C, rinsed with TBS, incubated with 0.3% H_2_O_2_ (in TBS) for 30 min at RT, and subsequently incubated with a rabbit anti-Ki67 antibody (1/4000; Abcam, Paris, France) at 4 °C overnight. The second day, the sections were rinsed with TBS-T and TBS, and incubated with the secondary antibody for 1 h at RT (1/800), with Vectastain ABC kit (Vector Laboratories, Burlingame, Ca, U.SA.) for 45 min at RT and with 3,3′-diaminobenzidine (DAB) for 10 min at RT in the dark. The sections were then rinsed successively in 70%, 90%, 100% ethanol, and Histoclear (National Diagnostics, VWR international SAS, Fontenay-sous-bois, France), after which they were mounted, and visualized using a microscope. Ki67 positive cells were counted using the plugin “cell counter” of ImageJ software.

### Statistical analysis

Data are represented as mean ± SEM. A Student’s t test was used for comparison between two groups. When comparing more than two conditions, a one-way analysis of variance (ANOVA) was applied, followed by Bonferroni *post hoc* testing for group-wise comparisons. In addition, a two-way ANOVA (treatment x genotype) followed by Bonferroni *post hoc* was used to investigate the effect of the chronic administration of fluoxetine in WT and KO mice. The level of significance was set as *P* < 0.05. Statistical analyses were performed with GraphPad Prism (version 6, GraphPad Software, San Diego, CA, USA). Fluoxetine effect on cell proliferation was compared between WT and KO mice, using multiple t-test with a Holm-Šídák correction for multiple comparisons.

## Supplementary information

Supplementary Dataset 1
