# Supplementary material for: 5-HTT independent effects of fluoxetine on neuroplasticity
Source: Sci Rep. 2019 Apr 19;9:6311. doi: 10.1038/s41598-019-42775-w (PMC6474908; doi:10.1038/s41598-019-42775-w)
Supplement: Supplementary file 1 — Supplementary Dataset 1 [file 41598_2019_42775_MOESM1_ESM.pdf]

5-HTT independent effects of fluoxetine on neuroplasticity

Marion J.F. Levy 1,2, Fabien Boulle 1,2, Michel-Boris Emerit 1, Corinne Poilbout 1, Harry W. Steinbusch 3, Daniel Van den Hove 3,4, Gunter Kenis 3, Laurence Lanfumey 1,2*

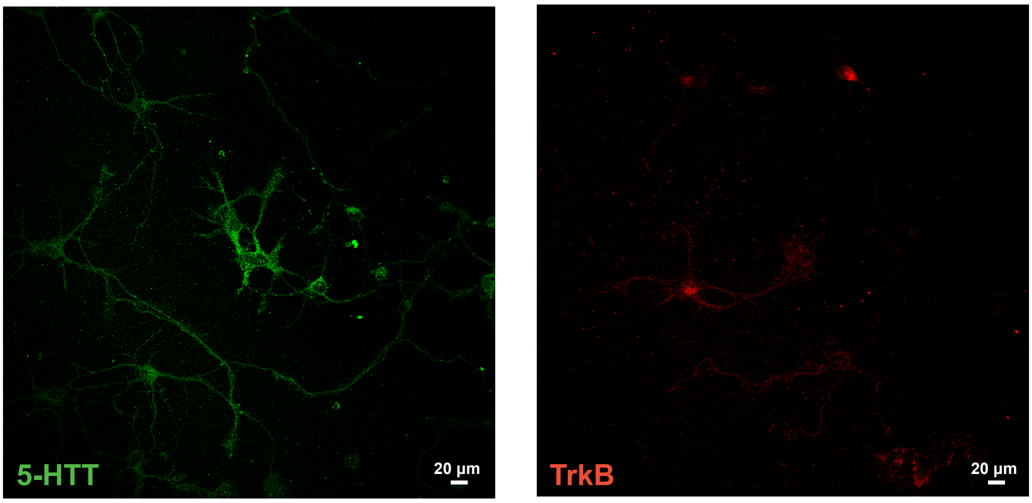

Supplementary Figure S1 : 5-HTT and TrkB immunofluorescence on non-treated cells from WT mice

Cells were fixated with 4% paraformaldehyde (Sigma Aldrich, Saint Quentin Fallavier, France) and 4% glucose (Sigma Aldrich, Saint Quentin Fallavier, France) for 15 minutes at 37 °C, rinsed with PBS, incubated in a blocking buffer (PBS 1X, BSA 2%, Donkey serum 3%, Triton X100, 0,1%) (Sigma Aldrich, Saint Quentin Fallavier, France and Thermo Fisher Scientific, Courtaboeuf, France) for 1 h at RT and subsequently incubated with primary antibodies goat anti-5-HTT (1/50, Santa Cruz Biotecnology Inc, Paso Robles, CA, USA) or rabbit anti-TrkB (1/50, Abcam, Cambridge UK) at 4 °C overnight. The second day, cells were rinsed with PBS and incubated either with a secondary antibody anti-rabbit alexa fluor 594 (1/500, Life Technilogies, Carlabad, CA, USA) for 1 h 30 for TrkB or first with a secondary antibody anti-goat biotinylated (1/200, Vector) for 1 h 30 and with streptavidin Alexa 488 (1/1000, Life Technilogies, Carlabad, CA, USA) for 1 h 30 for 5-HTT. Cells were then mounted with fluoromount and vizualized using a Leica TCS SP5 AOBS laser scanning confocal microscope.

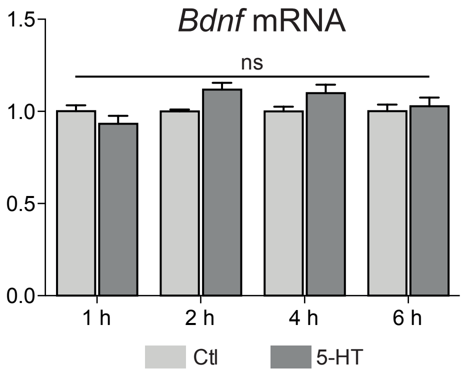

Supplementary Figure S2: Effects of serotonin (5-HT) on *bdnf* mRNA expression in cultured cortical neurons from C57BL/6J WT mice.

Cells were incubated for 1 h, 2 h, 4 h or 6 h with 10 µM of serotonin (Sigma Aldrich, Saint Quentin Fallavier, France) in cultured medium. Control medium or medium with serotonin were changed every 30 min. The expression of *Bdnf* was measured by RT-qPCR. No effect on *Bdnf* mRNA expression after serotonin incubation was observed. Two-way ANOVA. Data are expressed as mean + SEM of n=3.
Ctl: control; 5-HT: serotonin

Blot 1

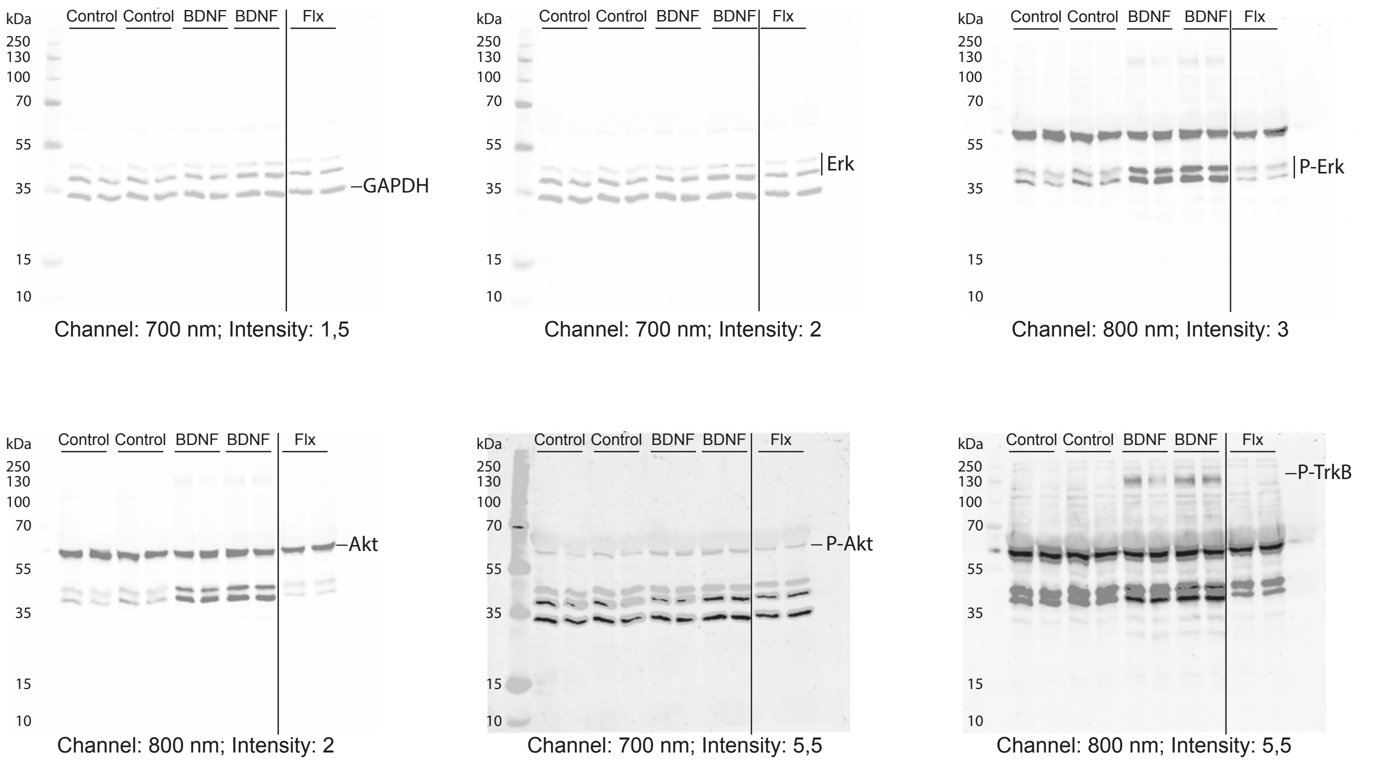
kDa
250
130
100
70
55
35
15
10
Control
BDNF
Flx
GAPDH
Erk
P-Erk
Akt
P-Akt
P-TrkB
Channel: 700 nm; Intensity: 1,5
Channel: 700 nm; Intensity: 2
Channel: 800 nm; Intensity: 3
Channel: 800 nm; Intensity: 2
Channel: 700 nm; Intensity: 5,5
Channel: 800 nm; Intensity: 5,5

Blot 2

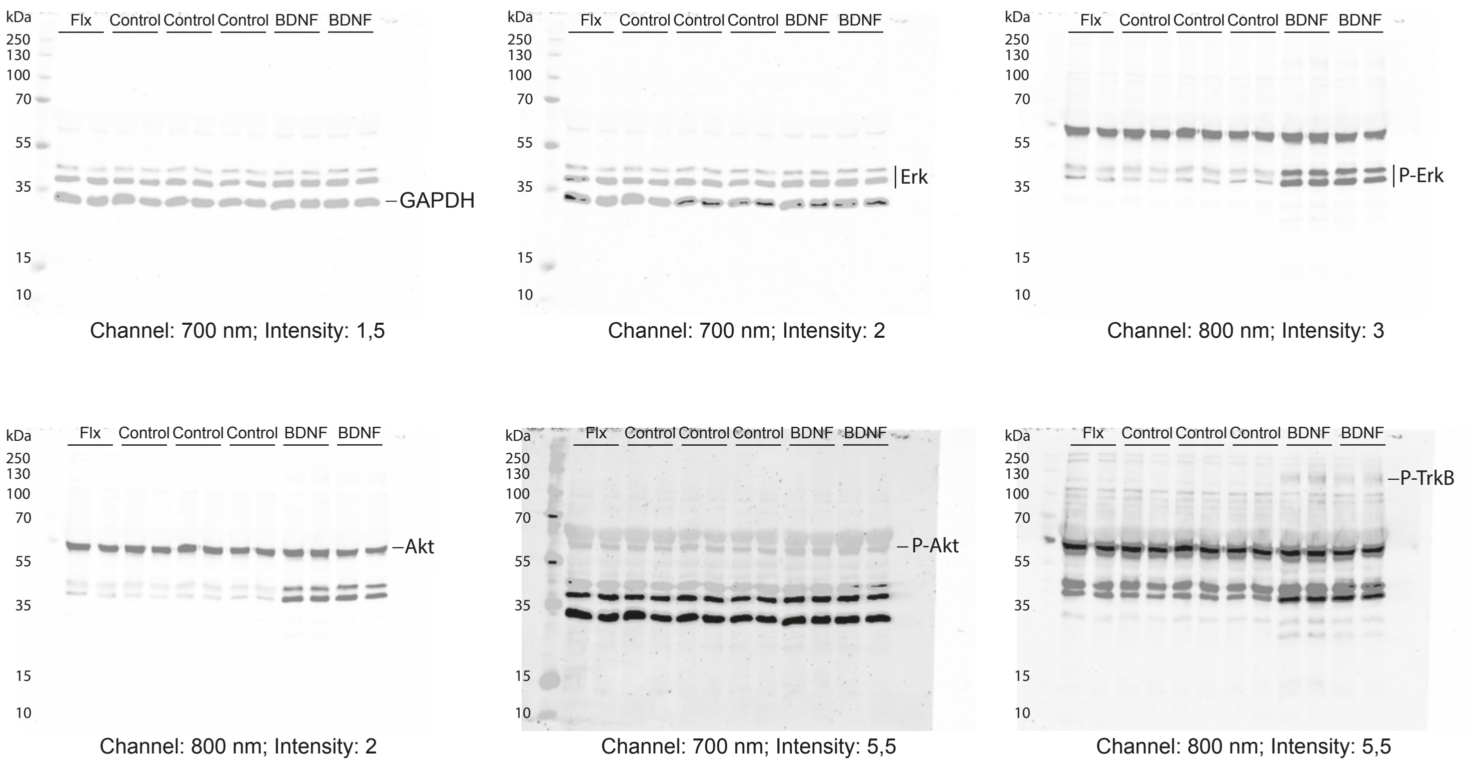
kDa
250
130
100
70
55
35
15
10
Flx
Control
BDNF
GAPDH
Erk
P-Erk
Akt
P-Akt
P-TrkB
Channel: 700 nm; Intensity: 1,5
Channel: 700 nm; Intensity: 2
Channel: 800 nm; Intensity: 3
Channel: 800 nm; Intensity: 2
Channel: 700 nm; Intensity: 5,5
Channel: 800 nm; Intensity: 5,5

Blot 3

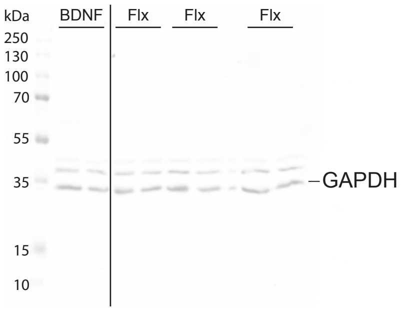

Channel: 700 nm; Intensity: 1,5

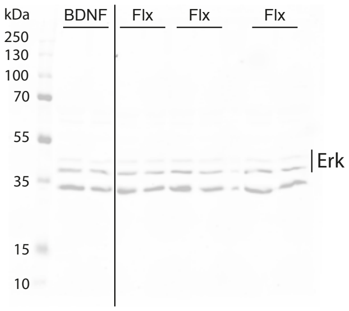

Channel: 700 nm; Intensity: 2

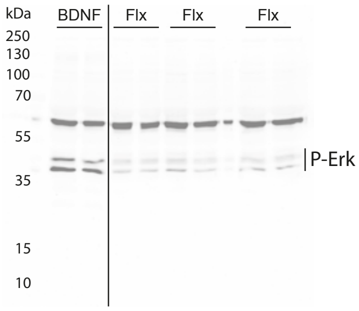

Channel: 800 nm; Intensity: 3

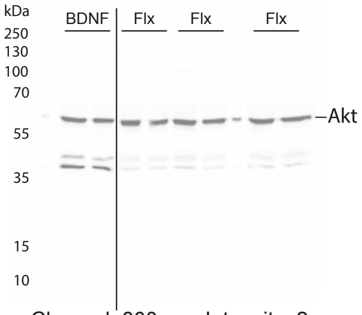

Channel: 800 nm; Intensity: 2

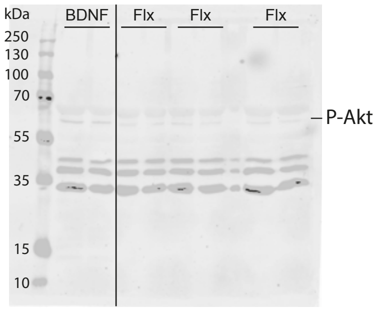

Channel: 700 nm; Intensity: 5,5

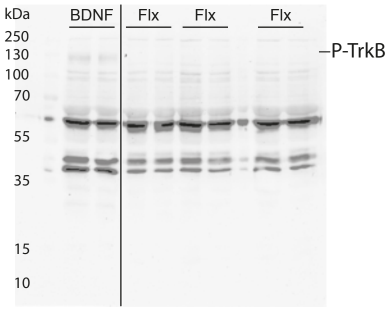

Channel: 800 nm; Intensity: 5,5

Blot 4

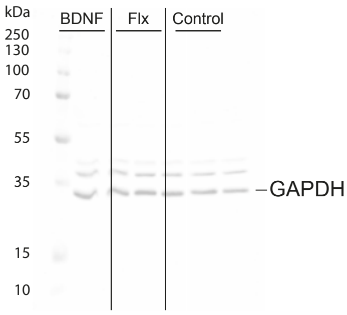

Channel: 700 nm; Intensity: 1,5

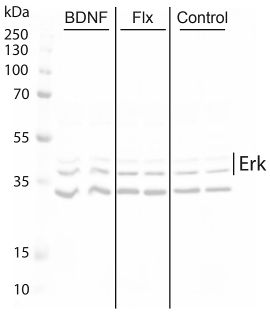

Channel: 700 nm; Intensity: 2

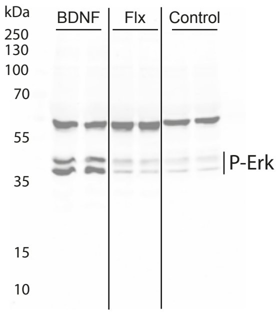

Channel: 800 nm; Intensity: 3

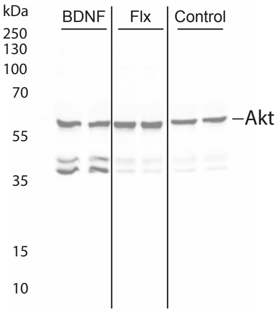

Channel: 800 nm; Intensity: 2

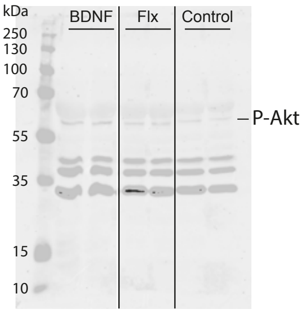

Channel: 700 nm; Intensity: 5,5

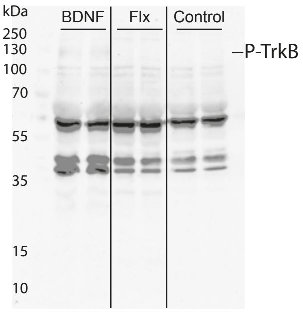

Channel: 800 nm; Intensity: 5,5

Blot 5

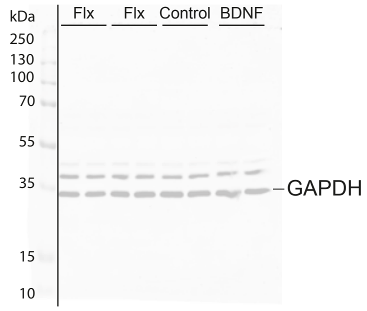

Channel: 700 nm; Intensity: 1,5

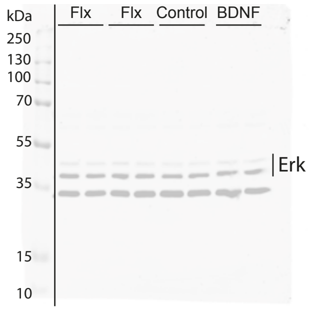

Channel: 700 nm; Intensity: 2

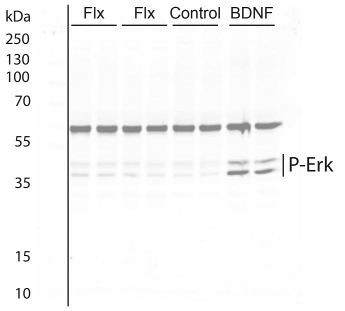

Channel: 800 nm; Intensity: 3

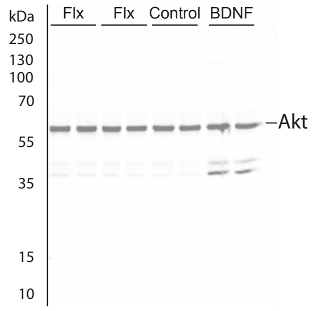

Channel: 800 nm; Intensity: 2

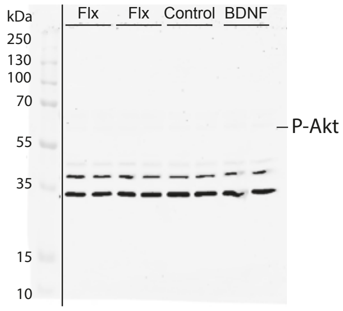

Channel: 700 nm; Intensity: 5,5

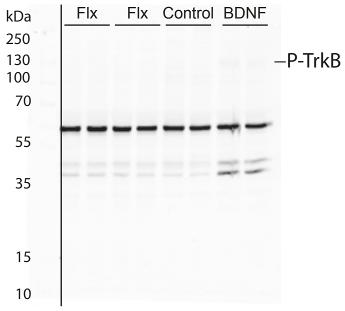

Channel: 800 nm; Intensity: 5,5

Supplementary Figure S3 : full-length blots

Full scanned blots for western blots shown in figures 2d.
Some blot images were cropped to merge mw markers with protein bands while removing samples unrelated to study which were ran on the same gel.

Flx: Fluoxetine
